# M. Powell Lawton Award Presentation

**DOI:** 10.1093/geroni/igaa057.3196

**Published:** 2020-12-16

**Authors:** Barbara Resnick

**Affiliations:** University of Maryland School of Nursing, Baltimore, Maryland, United States

## Abstract

Dr. Lawton focused his life work, starting in the 1960s, on improving the lives of older adults, particularly those with dementia. He did this at a time when care was custodial at best. He was innovative and initiated new thoughts about how to best care for individuals in institutional settings. He raised our awareness about the importance of the environment and the value of individualized care. We build on that work today. The focus of this 2019 Powell Lawton award presentation is on sharing my lifetime work, starting in 1972, on helping older adults, particularly those who are institutionalized and may have cognitive changes, maintain their functional ability and increase time in physical activity. Along with Dr. Lawton’s experiences, I and the teams I have worked with, have met challenges ranging from the use of Houdini vests to bed alarms and wheelchairs, to the persistent call out by caregivers to older adults-“don’t get up you might fall.” I will share information, resources and data from over a 20 year period developing and testing Function Focused Care, an approach to care that helps caregivers optimize function and increase physical activity among institutionalized older adults. Further, the process for how to disseminate and implement Function Focused Care across multiple settings and how to be a resilient researcher will also be reviewed. Participants will be able to take the findings and resources from this presentation and implement this approach into their own settings of care.

